# Quantifying the incremental value of deep learning: Application to lung nodule detection

**DOI:** 10.1371/journal.pone.0231468

**Published:** 2020-04-14

**Authors:** Theodore Warsavage, Fuyong Xing, Anna E. Barón, William J. Feser, Erin Hirsch, York E. Miller, Stephen Malkoski, Holly J. Wolf, David O. Wilson, Debashis Ghosh

**Affiliations:** 1 Department of Biostatistics and Informatics, Colorado School of Public Health, University of Colorado Anschutz Medical Campus, Aurora, CO, United States of America; 2 Department of Pulmonary Sciences and Critical Care Medicine, School of Medicine, University of Colorado Anschutz Medical Campus, Aurora, CO, United States of America; 3 Rocky Mountain Regional Veterans Affairs Medical Center, Aurora, CO, United States of America; 4 Department of Pulmonary and Critical Care Medicine, School of Medicine, University of Colorado Anschutz Medical Campus, Aurora, CO, United States of America; 5 Department of Community and Behavioral Health, Colorado School of Public Health, University of Colorado Anschutz Medical Campus, Aurora, CO, United States of America; 6 Division of Pulmonary, Allergy and Critical Care Medicine, Department of Medicine, University of Pittsburgh, Pittsburgh, PA, United States of America; University of Central Florida (UCF), UNITED STATES

## Abstract

We present a case study for implementing a machine learning algorithm with an incremental value framework in the domain of lung cancer research. Machine learning methods have often been shown to be competitive with prediction models in some domains; however, implementation of these methods is in early development. Often these methods are only directly compared to existing methods; here we present a framework for assessing the value of a machine learning model by assessing the incremental value. We developed a machine learning model to identify and classify lung nodules and assessed the incremental value added to existing risk prediction models. Multiple external datasets were used for validation. We found that our image model, trained on a dataset from The Cancer Imaging Archive (TCIA), improves upon existing models that are restricted to patient characteristics, but it was inconclusive about whether it improves on models that consider nodule features. Another interesting finding is the variable performance on different datasets, suggesting population generalization with machine learning models may be more challenging than is often considered.

## Introduction

Data captured for biological research has become increasingly complex. High-dimensional data, such as imaging data (MRI, CT, PET) and the so-called “omics” data (genomics, proteomics, metabolomics, etc.) have pushed the limits of traditional statistical analysis. These data can include many dimensions with complex interactions and nonlinearities. Adaptation of machine learning advancements helps to provide methods for reducing the dimensionality and modeling the interactions and nonlinearities.

Machine learning generally refers to methods that learn functions from data, either by identifying patterns with unsupervised data, or by finding features associated with a provided label in supervised learning. Methods include adaptations to existing methods, such as adding a regularization term to a regression model as in lasso or ridge regression. Other methods create more structure for nonlinearities, such as usage of decision trees. Multiple modeling techniques can be combined with ensemble methods, which combine predictions from multiple models for an overall prediction. Resampling techniques and iterative approaches are implemented to improve model performance, sometimes greatly increasing computational requirements. More sophisticated methods, such as deep learning models, have complex layered architectures with potentially millions of parameters. Choices of model architecture depend on the data and goal, and often several methods should be attempted. These methods have advanced biological data analysis, though implementation is not without challenges.

In response to Shah’s article, “Making Machine Learning Models Clinically Useful,” we present some first steps for assessing machine learning models using an incremental value framework [1]. As Shah notes, machine learning algorithms show promise to outpace clinicians in diagnostic accuracy with some types of data. However, implementation of these models in clinical settings is in early development.

Use of statistical models in clinical settings is already an established science [2]. Creation of diagnostic (detection of existing disease) or prognostic (predicting future state of disease) tools with statistical models can synthesize data into a quantitative risk. This results in more informed decisions from patients and physicians. Development of models has been conducted by many different research groups; however, transparent reporting of methods is often not consistent. In response, Moons et al. released the TRIPOD (Transparent Reporting of multivariable prediction model for Individual Prognosis or Diagnosis) statement, outlining recommendations for reporting prediction models [3]. As they note, modeling strategies are often poorly conducted, whether it be from small datasets, incorrect statistical modeling or inappropriate handling of missing data. Additionally, these models are often not validated on an external dataset. They suggest 22 items that should be reported to provide transparency to the data, the model development, model validation and any limitations.

Within the context of prediction modeling, assessing the incremental value of newly developed predictors, i.e. a newly discovered biomarker, is often the main question of interest. Several authors have summarized measures used for assessing incremental value and recommended best practices [4–6]. Steyerberg et al. [4] suggest using several measures of goodness of fit beyond the traditional measures (Brier score, concordance statistic (c-statistic) and goodness-of-fit statistics) including reclassification measures (net reclassification index (NRI) and integrated discrimination index (IDI)) and clinical usefulness measures (decision curve analysis or net benefit) [4]. They reiterate the need for external validation. We suggest developing machine learning models in the context of incremental value by assessing the machine learning model’s ability to enhance discrimination in conjunction with existing prediction models.

Implementing diagnostic or prognostic models with machine learning tools adds challenges to the existing framework of clinical prediction modeling. Machine learning models potentially have many parameters from which inference is limited. Knowing what features have been identified to classify data is often not possible, preventing the researcher from having complete understanding of the model. Features are learned from the data used for training and care must be taken that learned features will generalize to populations of interest, and not be specific to the trained data. Proper model development and validation with multiple external datasets is recommended to ensure generalization occurs. Here we present a case study for developing a machine learning model with imaging data in the domain of lung cancer research.

## Case study: Prediction of lung cancer from low-dose computed tomography (LDCT) scans

In response to the high mortality rate of lung cancer the National Lung Screening trial evaluated screening with low-dose computed tomography (LDCT) in high risk populations for earlier detection of disease [7]. It was found that with CT screening a 20% reduction in mortality was achieved when compared with standard chest x-rays [8]. Depending on baseline risk, screening is not always recommended because the cost and stress of false positives can outweigh the benefit of true positive findings. Radiologists detect lesions or nodules in the lung via CT scans, however cancer status cannot always be determined from the image data alone. Radiologists use protocols, such as Lung-RADS [9], to score a screening CT scan as positive or negative for malignancy, however uncertainty remains with many findings. Several risk prediction models have been developed to aid clinicians in determining cancer status. Varying levels of patient and nodule information are required. The Gould model requires nodule size as well as smoking information [10]. The McWilliams and Swensen (Mayo) model take into consideration additional nodule attributes such as edge characteristics and opacity as well as several additional patient variables such as history of cancer [11, 12]. The McWilliams model was designed to classify malignancy risk from a patient’s first CT screening for lung cancer. Swensen and Gould models were designed for newly discovered nodules. Analyzing imaging data is a domain where machine learning methods have greatly improved on previous methods, generating a suitable case study to assess the incremental value of machine analyzed CT scans to existing prediction models.

Computer aided diagnosis (CAD) is the process of using computer algorithms to aid clinicians in diagnosis of imaging data. Commercial software for detection and diagnosis of lung nodules has existed for some time [13]; however, starting in 2012, the implementation of deep learning to classify imaging data has greatly accelerated of image analysis algorithms. To further research in a field, public competitions are hosted to gather knowledge from the research community. Competitions in lung nodule detection and diagnosis include grand challenges such as LUNGx and LUNA16 in 2015 and 2016, respectively; the 2017 Kaggle data science bowl; and the ISBI 2018 (International Symposium on biomedical imaging) competition. It is important to note that the development of such competitions is very productive for collecting ideas on how to classify with a given domain; however, there are still major concerns about how they translate to clinical situations. Often the data being analyzed is simplified, such as removal of challenging or questionable nodules, and restriction to nodules above a larger size. Additionally, the metric of interest may differ from what is clinically useful, such as predicting the cancer status of the individual without reporting the number of nodules detected, the location and associated probability of cancer.

We used a public data set from The Cancer Imaging Archive (TCIA) to train our model, namely The Lung Image Database Consortium and Image Database Resource Initiative (LIDC-IDRI) [14–16]. This database was developed as a tool to aid the development of CAD for the lung nodule domain. It contains diagnostic or screening CT scans for 1010 individuals, and each scan has detailed annotations of nodule locations and segmentations. Three types of annotations are provided, the location of nodules less than 3 mm, the locations of suspicious regions the radiologist believes are not nodules, and the location, characteristics and segmentation of nodules from 3 to 30 mm in diameter. Main selection criteria included that the scan be of good resolution (distance between image slices less than or equal to 3 mm) and the presence of a nodule between 3 to 30 mm, though several scans only include annotations for non-nodule regions or nodules less than 3 mm. The LIDC-IDRI data mostly consisted of screening scans with nodule findings, however, diagnostic CT scans were also included. About 25% (~600 nodules) of the approximately 2600 nodules in the training dataset were larger than 8 mm, providing a reasonable training set for detecting nodules between 8 and 30 mm. It is important to note, that the database selection criteria were based on nodule and scan characteristics rather than a particular population of interest.

We developed a two-step approach to predicting cancer nodules in CT scans. We used one model to predict locations of suspected nodules, and then a second model to assign cancer probability to suspected nodules. State of the art nodule detection architectures were implemented; adaptations were made to a synthesis of a U-Net architecture [17] and Mixed Net architecture [18] as proposed by Nasrullah et al. [19]. Additional details are provided in the Appendix. Frequency receiver operating characteristic curves (FROC curves) are presented in the Appendix for the sensitivity and false positive rate of nodule detection for the initial model. For the detection of lung nodules, a higher sensitivity cutoff was used that captured 90% of nodules, with a 5.0 false positive per scan rate. The second model served to reduce the false positive rate, similar to online hard example mining, with cascading models. The second model was trained to classify cancer status of likely nodules, and used the radiologist marked likelihood of cancer as supervised labels. We note that the subjective radiologist likelihood as an outcome is a key limitation for our study.

We proceeded to apply the two models to two different cohorts of patients to determine a probability of cancer for each patient based on imaging data alone. Each cohort had information regarding the cancer status of an individual, so the model could be assessed on its ability to predict cancer in comparison to predictions from risk calculators. The first model was applied to each CT scan for a given cohort, producing locations of regions likely to be nodules and the second model was applied to obtain an associated probability of malignancy. The maximum probability of malignancy across detected regions was used to assess predictive accuracy and incremental value was assessed with respect to risk calculator probabilities.

Incremental value was assessed by combining the information from the image algorithm and the risk calculator. For each individual, we used Bayes’ rule to calculate a posterior probability of lung cancer, using the risk calculator as a prior probability and the imaging algorithm probability as new information. Following Ankerst et al., the posterior odds equals the likelihood multiplied by the prior odds [20]. Prior probabilities were transformed into odds and updated with a likelihood ratio from the image algorithm probabilities to produce a posterior odds. Posterior odds were transformed back to a probability. We compared the area under the curve (AUC) from receiving operating characteristic (ROC) curves calculated from the prior and posterior probabilities (as well as the imaging algorithm alone). We also calculated the integrated discrimination improvement (IDI) and show reclassification tables.

Posterior Odds = Likelihood Ratio*Prior Odds

$$\frac{P\left(Cancer\right|X,Y)}{P(No\;Cancer|X,Y)}\;=\;\frac{P(Y|Cancer,X)}{P(Y|No\;Cancer,\;X)}\;\times \;\frac{P(Cancer|X)}{P(No\;Cancer|X)}$$

where X are patient covariates and nodule features used in developing risk models and Y is the CT scan image data.

For usage of the Colorado Lung Nodule Cohort, the Colorado Multiple Institutional Review Board approved our access for research by declaring the study involves minimal risk and met requirements for a full waiver of consent and HIPAA Authorization. Access of the PLuSS nodule cohort was provided by PLuSS providers through a collaboration between the Colorado SPORE research group and the Pittsburgh PLuSS study.

### Colorado Lung Nodule

**Fig 1:**
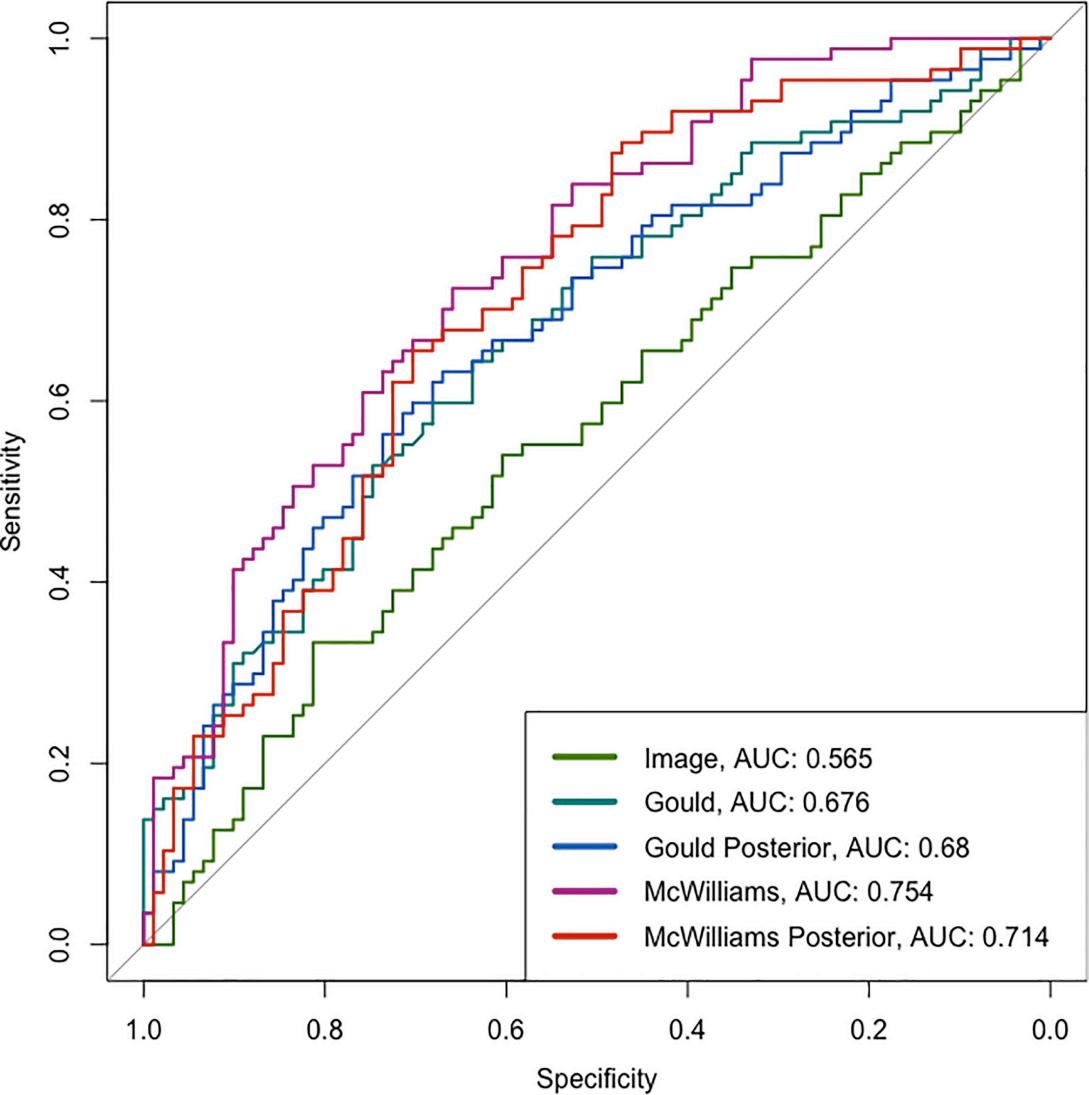
Receiver operating characteristic curves and associated AUC are shown for the image model predictions and risk calculator predictions on Colorado Lung Nodule Cohort. The model Image refers to our algorithm, the Gould and McWilliams are risk calculators and the posterior models are the Gould and McWilliams models updated with information from the image model.

Our first cohort, The Colorado Lung Nodule Cohort, consisted of individuals referred to pulmonologists, oncologists, or thoracic surgeons for evaluation of incidentally identified lung nodules on CT scans [21]. Individuals were required to have one or more lung nodules between 8 and 30 mm to be included and were excluded if nodules had solid calcification, or the patient had a low life expectancy (< 6 months) or known cancer. We obtained CT scans for 298 patients; the image model was not predictive in four, and clinical data was only present for a subset resulting in 178 images for the final dataset. Patient-level data was available to calculate the McWilliams and Gould risk scores for assessing incremental value. Despite similarities with the training data, the model did not perform well on the Colorado Lung Nodule Cohort. The image model was only slightly better than random chance with an AUC of 0.565 (Fig 1). See the reclassification tables (see Table 1) for groupings on which groups of individuals were reclassified. We used the McWilliams risk calculated as a prior and updated the risk score with the image predictions for a posterior probability of lung cancer. There were more individuals with a low score in the initial model. The individuals with a higher prior were more frequently reclassified. The integrated discrimination improvement (IDI) for the image data added to the McWilliams risk model predictions was 0.033 (95% CI: (-0.031, 0.097); p-value: 0.311), showing minimal improvement. The IDI for improvement of the Gould model was less significant, with a value of 0.029 (95% CI: (-0.033, 0.091); p-value: 0.364). Visual examination of the likely malignant regions detected in the cohort did not always appear to include a nodule between 8 to 30 mm, suggesting the model was either not finding the nodule of interest, or finding another region as more suspicious than the nodule.

**Table 1:** Reclassification of nodules in Colorado Lung Nodule Cohort from updating the McWilliams risk prediction model with the Image model. The initial model is the McWilliams prediction model, the updated model is the McWilliams prediction score updated with Image model prediction with Bayes’ rule. More improvement is seen in the event class with probabilities 0.4 to 0.8, compared to the non-event class.

**Nonevents (n = 91)**						
** **	**Updated model**					** **
Initial Model	[0,0.2)	[0.2,0.4)	[0.4,0.6)	[0.6,0.8)	[0.8,1]	% reclassified
[0,0.2)	36	12	0	2	0	28
[0.2,0.4)	6	6	4	4	3	74
[0.4,0.6)	3	3	1	5	3	93
[0.6,0.8)	0	0	0	0	3	100
[0.8,1]	0	0	0	0	0	NA
Events (n = 87)						
	Updated model					
Initial Model	[0,0.2)	[0.2,0.4)	[0.4,0.6)	[0.6,0.8)	[0.8,1]	% reclassified
[0,0.2)	10	5	1	1	0	41
[0.2,0.4)	4	8	8	3	1	67
[0.4,0.6)	2	2	6	6	14	80
[0.6,0.8)	0	3	1	5	7	69
[0.8,1]	0	0	0	0	0	NA

### Pittsburgh Lung Screening Study

Next, we applied the model to several lung nodule datasets from the Pittsburgh Lung Screening Study (PLuSS) cohort [22] and Cooper cohort. For the PLuSS cohort, 3,642 individuals at high risk for lung cancer (ages 50–79, current of former smoker, no current cancer diagnosis) were screened with low-dose CT for lung nodules. The Cooper cohort consisted of individuals identified with clinical CT scans (these scans used higher radiation level than screening CT scans and thus had higher image quality), though inclusion was not restricted by nodule size. The pilot dataset was pulled from the PLuSS cohort, and included 51 cancer and 54 non-cancer cases, though nine non-cancer cases were removed for no nodule or patient level data. The validation dataset, consisted of 130 images from the PLuSS cohort, and 70 images from the Cooper cohort, resulting in a total of 200 images, 100 cancer cases and 100 non-cancer cases, selected according to available case or randomly from remaining sets. This dataset more closely matched the training data since it was a screening cohort without restriction on nodule size. Limited clinical data was available, only allowing for the assessment of incremental value with respect to the Gould prediction model. Some data was missing for the Gould model covariates in the dataset. In addition, individuals were either removed when data failed to process, or multiple imputation with Rubin’s rules was used to obtain an estimate of the IDI [23]. The image model performed better on these datasets.

**Fig 2:**
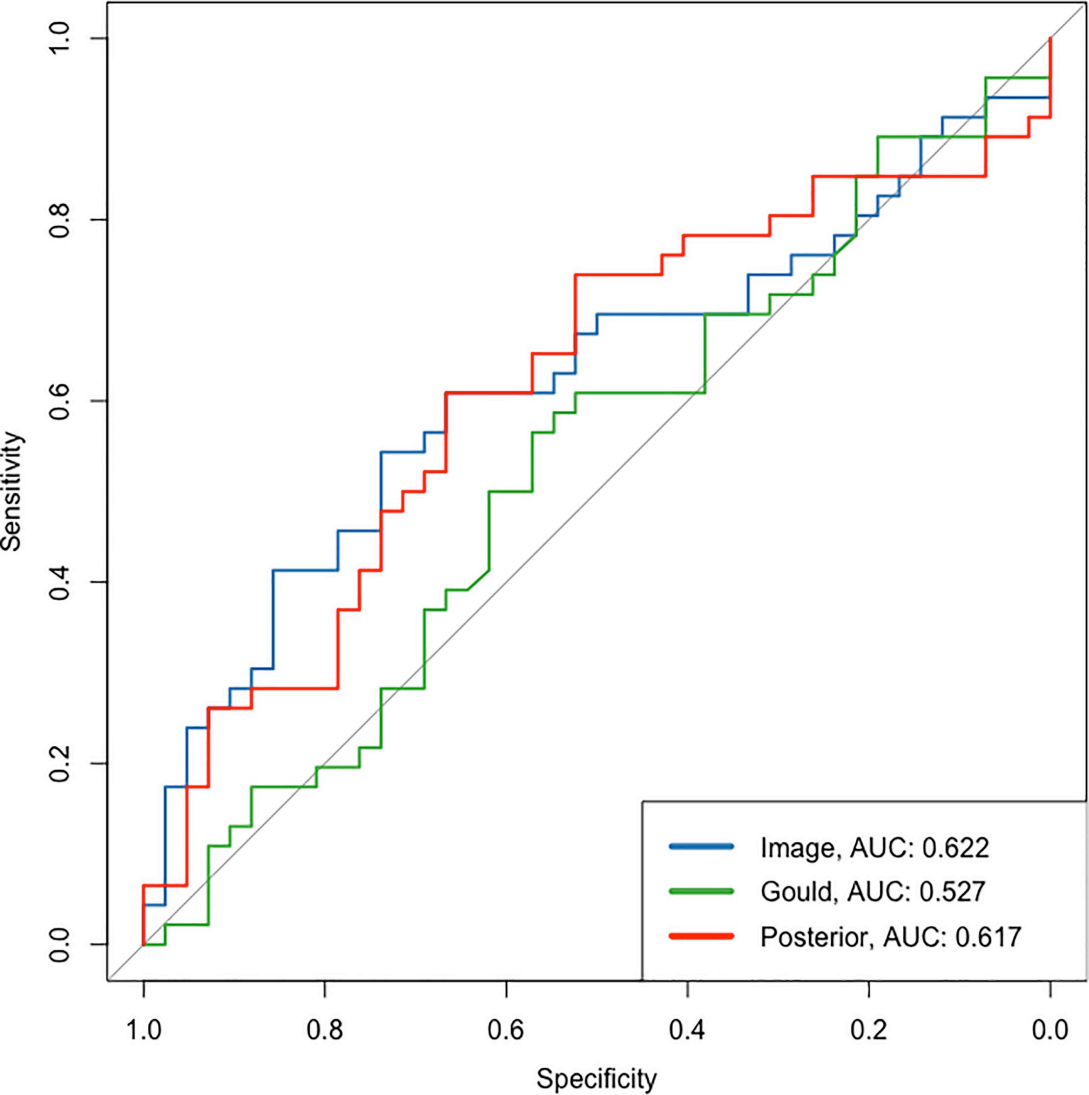
Receiver operating characteristic curves and associated AUC are shown for the Image model and Gould model applied to the pilot PLuSS dataset. The Image model refers to our model that only used the CT data for predictors, the Gould model is from the Gould risk calculator and the posterior model is the Gould model updated with information from the Image model.

For the pilot dataset using the PLuSS cohort, the image model did not predict any nodules for several scans, resulting in a total of 46 cases and 42 non-cases with prior and posterior predicted probabilities of cancer. The Gould prediction model had an AUC of 0.527, the image model had an AUC of 0.622, and the posterior probability after updating the Gould model with the image model had an AUC of 0.617 (Fig 2). This improvement is also shown in the IDI, which was 0.076 (95% CI: 0.006–0.145; p-value: 0.033). For most individuals, the probability of malignancy was increased after updating the model with the image model, but the event class consistently had a higher percentage of individuals classified upwards compared to the non-event individuals; see the reclassification table (Table 2) for further detail.

**Table 2:** Reclassification table for the pilot PLuSS datasets. The initial model is based on the Gould prediction model, the updated model incorporates the predicted probabilities from our Image model into the Gould predictions. More individuals are reclassified to a higher probability class in the event group compared to the nonevent group.

**Nonevents (n = 42)**						
	**Updated model**					
Initial Model	[0,0.2)	[0.2,0.4)	[0.4,0.6)	[0.6,0.8)	[0.8,1]	% reclassified
[0,0.2)	0	1	0	0	0	100
[0.2,0.4)	5	8	2	0	0	47
[0.4,0.6)	1	4	6	2	0	54
[0.6,0.8)	0	2	2	4	1	56
[0.8,1]	0	1	1	1	1	75
Events (n = 46)						
	Updated model					
Initial Model	[0,0.2)	[0.2,0.4)	[0.4,0.6)	[0.6,0.8)	[0.8,1]	% reclassified
[0,0.2)	1	0	1	0	0	50
[0.2,0.4)	5	4	6	1	0	75
[0.4,0.6)	1	2	8	2	1	43
[0.6,0.8)	0	0	2	3	3	62
[0.8,1]	0	0	0	2	4	33

**Fig 3:**
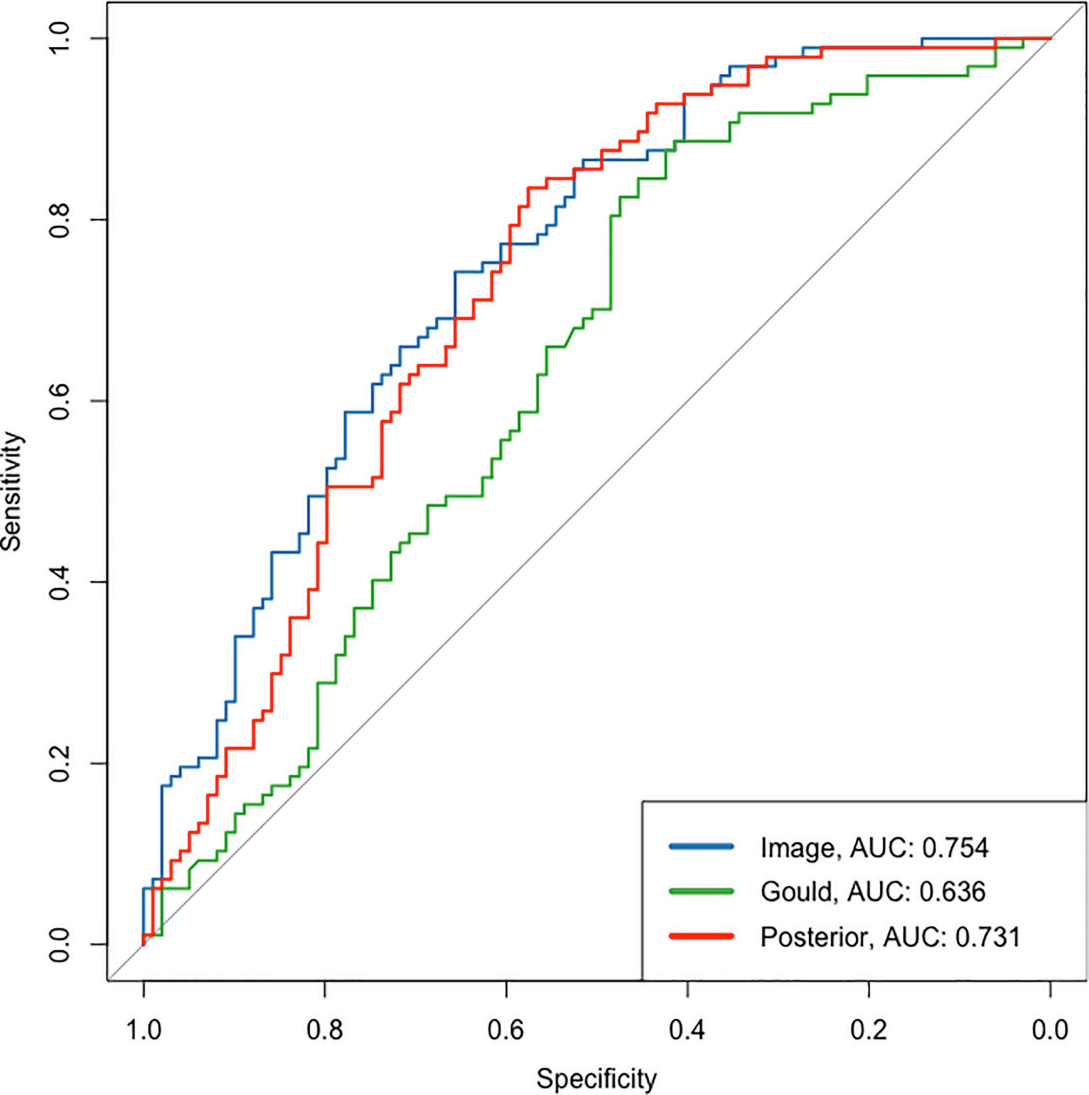
Receiver operating characteristic curves and associated AUC are shown for the Gould prediction model on its own, the image model on its own and the incremental value of the Image model incorporated into the Gould model which we called the posterior model. This is a sample of one of five imputed datasets.

In the validation dataset the image model did not predict any suspicious regions for five CT scans and were removed. 70 individuals were missing information on years since quit smoking. Multiple imputation with chained equations (mice) was used to create 5 datasets on which incremental value was assessed and the estimates combined using Rubin’s rules [23]. The mean AUC of the prediction model was 0.653 for the imputed datasets, the Image model had an AUC of 0.754, and the mean AUC for the posterior was 0.743. Improvement is also exhibited with an average IDI value of 0.148 (95% CI: 0.103–0.192; p-value < .0001) across the imputed datasets. Estimates and standard errors across imputed datasets were combined with Rubin’s rules to calculate a p-value [23]. Example ROC plots (Fig 3) and reclassification table (see Table 3) from a randomly chosen imputation dataset are shown below. The image model performed considerably better on the PLuSS validation dataset compared to the PLuSS pilot dataset.

**Table 3:** Reclassification table on PLuSS validation dataset with Gould prediction model as initial model, and updated model with Image model probability incorporated into the Gould risk prediction score. Although both non-events and event groups were generally classified to higher probability classes, this effect was more pronounced in the event group. This is a sample of one of five imputed datasets.

Nonevents (n = 97)						
	Updated model					
Initial Model	[0,0.2)	[0.2,0.4)	[0.4,0.6)	[0.6,0.8)	[0.8,1]	% reclassified
[0,0.2)	45	3	0	0	0	6
[0.2,0.4)	9	4	4	1	0	78
[0.4,0.6)	3	9	6	5	0	74
[0.6,0.8)	0	1	2	0	2	100
[0.8,1]	0	0	0	2	1	67
Events (n = 99)						
	Updated model					
Initial Model	[0,0.2)	[0.2,0.4)	[0.4,0.6)	[0.6,0.8)	[0.8,1]	% reclassified
[0,0.2)	23	6	3	1	0	30
[0.2,0.4)	4	6	4	0	0	57
[0.4,0.6)	0	1	4	4	9	78
[0.6,0.8)	0	0	2	8	15	68
[0.8,1]	0	0	0	0	9	0

## Discussion

The problem of lung nodule detection and classification presents a valuable case study for the complications involved in developing and evaluating machine learning models for diagnostic and prognostic use in clinical settings. Our model was trained on a dataset of over 1000 CT scans, with over 2600 segmented lung nodules, developed with the intent to represent the distribution of lung nodules for developing machine learning technologies. We then used the model to predict locations of lung nodules and associated probability of cancer on several cohorts. We compared these predictions to existing lung nodule risk calculators in the context of incremental value. The performance of the model on each dataset varied greatly. The Colorado Lung Nodule Cohort, which consisted of incidentally identified nodules larger than 8 mm was most dissimilar to our training data, which was largely screening CT scans including nodules 3 to 30 mm, with only 25 percent nodules being larger than 8 mm. From review of the images of the most likely cancer regions, many appeared to be nodules larger than 8 mm, but some did not, suggesting the two-model process was not always succeeding to classify the same lesion for which the patient was entered into the cohort. Additional steps could have included a transfer learning step, to tune our trained model for detection of nodules 8 mm or larger, however, locations of nodules in the Colorado Lung Nodule Cohort were not available. Our intent was to build a model to detect and classify any nodule between 3 and 30 mm, and one quarter of the nodules in the LIDC dataset were above 8 mm, yet the model did not generalize to this validation dataset. Additionally, the Colorado Lung Nodule Cohort consists of individuals incidentally identified, rather than through screening. Inherent differences in these populations could be a factor in the poor generalization. Detailed assessment of these issues is challenging given that the features used for classification are contained in the model parameters. Such generalization errors may not be obvious without extensive validation.

The model performed better on the two datasets from Pittsburgh, though still with considerable variability. The Image model applied to the pilot dataset had a lower overall AUC of 0.622, compared to the validation dataset with an AUC of 0.754. Several of the pilot dataset CT scans were lower resolution and sometimes had a grainy appearance, which may have contributed to this discrepancy. Interestingly the Gould model was not predictive for the pilot dataset, which is surprising given the Gould model was developed for newly discovered nodules which is consistent with the pilot dataset. The validation dataset contained higher quality images given the mixture of both screening and clinical CT scans. This was more similar with our training data (LIDC-IDRI) which also contained both screening and clinical scans. Most of the LIDC-IDRI scans had a distance between slices of less than or equal to 2.5 mm, and from general observation had clearer resolution than many of the Colorado Lung Nodule cohort scans and pilot dataset scans. This suggests that image resolution and scan quality also could have been a significant factor in the variable performance. The validation dataset generally appeared to have the best image quality of our validation datasets and also had the best performance with regard to predictive ability with the Image model.

Often the accuracy of a machine learning model is presented by only comparing its effectiveness to contemporary measures. Presentation of effectiveness in the context of incremental value more clearly represents whether a machine learning model can improve on established clinical tools or approaches. The implementation of a machine model on lung nodules ideally automatically synthesizes the nodule characteristics such as spiculation and mixed solidity, into a single prediction score. For our implementation in the Pittsburgh datasets, the image data outperformed the Gould prediction model, and using information from both obtained improved results. However, it is not immediately clear whether the Imaging model can add to the McWilliams prediction model. It may be that existing risk prediction scores with consideration of nodule size, spiculation, and solidity, could be just as good as a machine learning model. It should be considered how much additional information may exist in the image data compared to what is already being captured. In some cases, a machine may be able to extract untapped information, but this isn’t guaranteed. Assessing the degree of improvement is central to presenting a compelling argument for adoption of such models.

Finally, we recognize our example was limited, especially with regard to our measure of malignancy in the training data. Using a subjective radiologist score is similar in function to using radiologist identified features in a risk prediction calculator, such as including nodule size and solidity in the McWilliams and Swensen prediction models. Obtaining complete high-quality datasets for developing machine learning models is very labor intensive. A dataset with nodule-specific verified malignancy could be used to identify features that may not be apparent to a radiologist and could exhibit improved discrimination beyond what was estimated here.

## Supporting information

S1 Appendix (DOCX)

S1 Fig Frequency receiver operating characteristic (FROC) curves are presented for the nodule detection model. A cutoff of 0.995 was used to identify pixels of likely nodules. Using clusters of at least 1 pixel above .955, had a sensitivity of 90% and false positive rate of 5.0; increasing cluster size reduced the sensitivity and false positive rate as shown in the graph.
(TIFF)
