# Supplementary material for: Quantifying the incremental value of deep learning: Application to lung nodule detection
Source: PLoS One. 2020 Apr 14;15(4):e0231468. doi: 10.1371/journal.pone.0231468 (PMC7156089; doi:10.1371/journal.pone.0231468)
Supplement: S1 Appendix — (DOCX) [file pone.0231468.s001.docx]

# **Appendix**

All data analysis was conducted using Python 3.6. TensorFlow 1.12 was used for training of deep learning models, using two GTX 1070 Ti graphics processing units. CT scans were imported and decrypted using the pydicom package. Once imported, scans were isometrically rescaled, setting pixel distances to 1 mm. All images were processed as 3D arrays. Pixel intensities were rescaled to Hounsfield units, according to scan descriptions, followed by standardization to region between -1 and 1 with an approximate mean of 0. Segmentation of lungs was completed for use with predictions, though raw image data was used for training.

The latest architectures for nodule detection were used as suggested by Nasrullah et al. [19]. Nasrullah adapted ideas from Mixed nets and U-Net. We similarly adapted Nasrullah’s suggested model by increasing the number of filters in mixed net blocks as suggested by the designers of Mixed networks, Wang et al. [18]. See Table 4 for model architecture details. Image patches of 96x96x96 pixels were used for training with pixel wise segmentations as ground truth. Data for training was selected according to a nodule being found randomly within the image region. Images were also rotated and flipped for 16 different orientations for each segmented nodule. 1/8^th^ of the patients in the training dataset were selected for testing set. Predictions were generated at each pixel for probability of nodule vs. non-nodule. Drop-out was implemented to increase generalization and batch normalization was implemented to speed training and reduce covariate shift.

**S1 Table: Model Architecture**

| **Convolution Stage** | **Output** | **Weights** | **Repetitions** |
| --- | --- | --- | --- |
| Initial input size | 96 x 96 x 96, 96 | 3 x 3 x 3, 96 |  |
| Mix Net block 1 | 96 x 96 x 96, 160 | 1 x 1 x 1, 32 | x4 |
|  |  | 3 x 3 x 3, 32 |  |
| Dimension reduction | 96 x 96 x 96, 96 | 1 x 1 x 1, 96 |  |
| 3D pooling | 48 x 48 x 48, 96 |  |  |
| Mix Net block 2 | 48 x 48 x 48, 160 | 1 x 1 x 1, 32 | x4 |
|  |  | 3 x 3 x 3, 32 |  |
| Dimension reduction | 48 x 48 x 48, 96 | 1 x 1 x 1, 96 |  |
| 3D pooling | 24 x 24 x 24, 96 |  |  |
| Mix Net block 3 | 24 x 24 x 24, 160 | 1 x 1 x 1, 32 | x4 |
|  |  | 3 x 3 x 3, 32 |  |
| Dimension reduction | 24 x 24 x 24, 96 | 1 x 1 x 1, 96 |  |
| Deconvolution and concatenation | 48 x 48 x 48, 192 | 3 x 3 x 3, 96 |  |
| Dimension reduction | 48 x 48 x 48, 96 | 1 x 1 x 1, 96 |  |
| Mix Net block 4 | 48 x 48 x 48, 160 | 1 x 1 x 1, 32 | x4 |
|  |  | 3 x 3 x 3, 32 |  |
| Dimension reduction | 48 x 48 x 48, 96 | 1 x 1 x 1, 96 |  |
| Deconvolution | 96 x 96 x 96, 96 | 3 x 3 x 3, 96 |  |
| Dimension reduction | 96 x 96 x 96, 96 | 1 x 1 x 1, 96 |  |
| Dimension reduction | 96 x 96 x 96, 32 | 1 x 1 x 1, 32 |  |
| Dimension reduction and soft-max | 96 x 96 x 96, 2 | 1 x 1 x 1, 2 |  |

Model was trained using backpropagation with stochastic gradient descent, with cross entropy as the loss function. Due to the large class imbalance in each image, a pixel-wise weight matrix for each image was implemented, upweighting nodule pixels to have comparable weight to the non-nodule pixels. A cutoff of .995 was determined to have the optimal sensitivity and false positive rate for the initial model (to maximize sensitivity) for detecting nodules, the final model had an average sensitivity of 90% and false positive rate per scan of 5.0 in the test patient set. A cutoff of .999 was determined to have a sensitivity of 83% and false positive rate of 2.1, however, this sensitivity was considered too low for the initial selection model. The final model served to further reduce false positives, similar to a hard example mining approach or cascading models.

For the model to predict likelihood of cancer, a similar architecture was used except with the final layers consisting of more pooling and several fully connected layers to produce a binary result of likelihood of cancer vs non-cancer as opposed to pixel-wise probabilities. The final model also only consisted of patches of likely nodule regions of size 24x24x24 pixels. The likelihood of malignancy was provided by four radiologists on the Likert scale for each nodule. These values were averaged and rescaled between 0 and 1 and served as supervised labels for nodule malignancy probability. Cross entropy loss with stochastic gradient descent was again implemented during training. In addition to known nodules, regions that were not nodules but predicted to be a nodule based on the location model, were included in the training data with a score of 0.
