# Supplementary figures and images for: Quantifying the incremental value of deep learning: Application to lung nodule detection
Source: PLoS One. 2020 Apr 14;15(4):e0231468. doi: 10.1371/journal.pone.0231468 (PMC7156089; doi:10.1371/journal.pone.0231468)

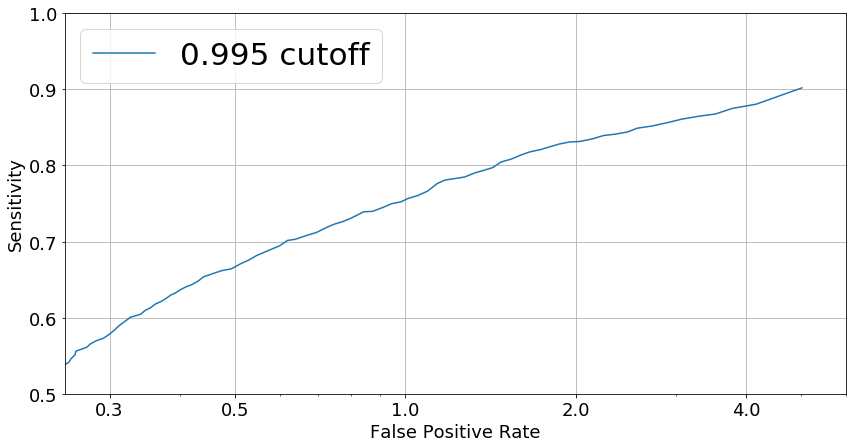

Supplement: S1 Fig — A cutoff of 0.995 was used to identify pixels of likely nodules. Using clusters of at least 1 pixel above .955, had a sensitivity of 90% and false positive rate of 5.0; increasing cluster size reduced the sensitivity and false positive rate as shown in the graph. (TIFF) [file pone.0231468.s002.tiff]
